# Enabling the assessment of trauma-induced hemorrhage via smart wearable systems

**DOI:** 10.1126/sciadv.abb1708

**Published:** 2020-07-22

**Authors:** Jonathan Zia, Jacob Kimball, Christopher Rolfes, Jin-Oh Hahn, Omer T. Inan

**Affiliations:** 1 Department of Electrical and Computer Engineering, Georgia Institute of Technology, Atlanta, GA 30332, USA.; 2 Emory University School of Medicine, Atlanta, GA 30322, USA.; 3 Translational Training and Testing Laboratories Inc., Atlanta, GA 30313, USA.; 4 Department of Mechanical Engineering, University of Maryland, College Park, MD 20742, USA.

## Abstract

As the leading cause of trauma-related mortality, blood loss due to hemorrhage is notoriously difficult to triage and manage. To enable timely and appropriate care for patients with trauma, this work elucidates the externally measurable physiological features of exsanguination, which were used to develop a globalized model for assessing blood volume status (BVS) or the relative severity of blood loss. These features were captured via both a multimodal wearable system and a catheter-based reference and used to accurately infer BVS in a porcine model of hemorrhage (*n* = 6). Ultimately, high-level features of cardiomechanical function were shown to strongly predict progression toward cardiovascular collapse and used to estimate BVS with a median error of 15.17 and 18.17% for the catheter-based and wearable systems, respectively. Exploring the nexus of biomedical theory and practice, these findings lay the groundwork for digital biomarkers of hemorrhage severity and warrant further study in human subjects.

## Teaser

Assessing the mechanical function of the heart using wearable sensors may soon enable robust, data-driven treatment of hemorrhage.

## INTRODUCTION

For patients suffering from trauma injury, timely and appropriate care is essential for preventing potentially fatal complications. One such complication is low blood volume due to exsanguination, known as absolute hypovolemia. As the leading cause of death in both military and civilian trauma cases, it is incumbent upon health care providers to rapidly assess the severity of hypovolemia and titrate care appropriately (*1*, *2*).

Triaging and managing absolute hypovolemia, however, are often intractable with traditionally available vital signs such as heart rate (HR) and blood pressure (BP): HR is a nonspecific feature of cardiac function, and BP is subject to several compensatory mechanisms that preserve arterial pressure (*3*). Further complicating matters is the interpatient variability in tolerance to hypovolemia—namely, a fixed volume of blood loss may result in varying levels of cardiovascular decompensation across the diverse patient population. In the context of clinical care, it is therefore necessary to determine a patient’s progression toward cardiovascular collapse, also known as blood volume status (BVS), rather than simply the volumetric extent of exsanguination.

Toward the development of clinical tools for rapidly assessing trauma injury, prior studies have identified a variety of physiological features that correlate with changes in blood volume. However, the correlation of a variable with a particular outcome of interest in a patient does not imply that the variable is predictive of the outcome in other patients who may have different physiological set points. Creating effective clinical tools for BVS assessment therefore requires a greater understanding of not only the physiological manifestations of hemorrhage but also the consistency and predictive value of such responses. Specifically, to pave the way toward future devices and algorithms for automated hemorrhage management, a deeper investigation of physiological parameters that could be measured with wearable sensing modalities is needed.

Perhaps the most notable use of wearable sensing for this task has been the use of arterial pressure waveforms derived from photoplethysmography to estimate the patient’s capacity to compensate for reductions in blood volume (*4*). This approach has shown success in predicting the individual-specific risk of cardiovascular collapse during progressive hypovolemia; however, it does not incorporate the effects of hypovolemia on the electrical and mechanical aspects of cardiac function. By developing systems that obtain a holistic, multifaceted view of cardiovascular health and performance, we may enable more robust clinical tools for trauma management, achieving a synergistic effect with prior work in this field.

**Fig. 1:**
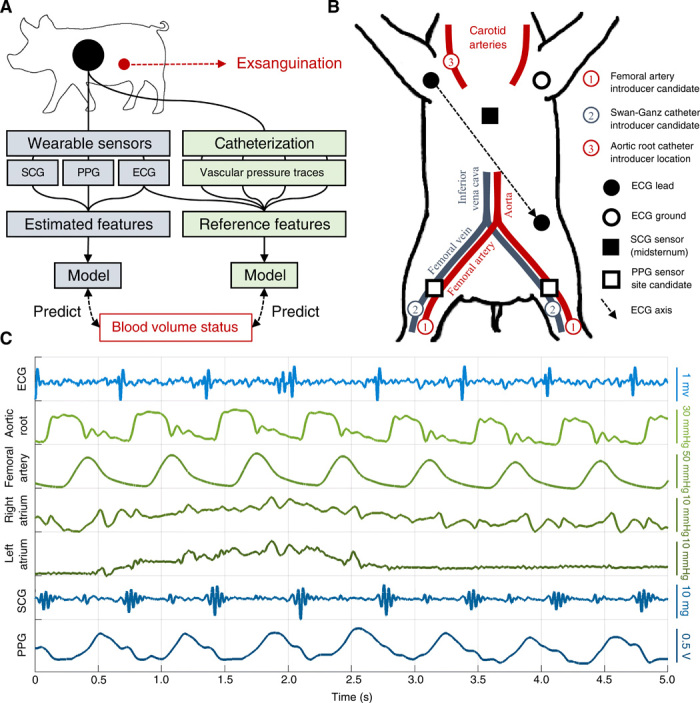
Overview of methods and data. (**A**) Overall method used in this work. A wearable sensing system (blue) was used to develop a globalized model of BVS assessment, which was then compared with a catheter-based gold standard system (green). The catheter-based system directly measured aortic root, femoral artery, right atrial, and left atrial (via pulmonary capillary wedge) pressures. (**B**) Diagram of sensor placement during experiment. Each catheter was placed in one of the indicated candidate locations for each subject depending on artery and vein accessibility. The photoplethysmogram (PPG) sensor was then placed on the most accessible femoral artery branch. (**C**) Example traces obtained from wearable- (blue) and catheter-based (green) sensors during a single respiration cycle.

Accordingly, to further enable BVS assessment, the goals of this work were (i) to elucidate the manner in which progressive hemorrhage affects common electrical, mechanical, and vascular features of cardiac function, and (ii) use these insights to develop a globalized model for the assessment of BVS for deployment on wearable sensing systems. As shown in Fig. 1A, multiple modalities of wearable sensors were used to monitor cardiovascular function in a porcine model (*n* = 6) undergoing an exsanguination protocol (i.e., volume depletion). During this time, vascular pressures were recorded concurrently using direct arterial and venous catheterization, which served as a gold standard reference for the performance of the wearable sensors. A variety of physiological features, which have been shown to covary with blood volume, were then derived from both the wearable- and catheter-based systems. Using a machine learning approach, the most consistent physiological responses to hemorrhage were identified and used to develop a globalized model of BVS assessment.

While the methods in this work are preclinical in nature—and will thereby require further investigation before clinical application —the results detailed below represent an important milestone toward the development of a digital biomarker of hemorrhage severity and, ultimately, the data-driven management of trauma injury. Specifically, prior knowledge is advanced by (i) directly comparing the utility of physiological features, including not only common features such as HR and BP but also less commonly explored features such as those derived from cardiomechanical signals, for BVS assessment; (ii) synthesizing data from multiple sensing modalities into an integrated system for this task; (iii) demonstrating the potential clinical utility of this approach by assessing progression toward cardiovascular collapse rather than raw blood volume loss; (iv) leveraging a volume-depletion animal model to elucidate this new understanding of physiological features of hemorrhage; and (v) exploring the inherent challenges in BVS assessment and providing concrete avenues for future development.

## RESULTS

### Monitoring system and experimental design

To enable the assessment of cardiovascular function using wearable sensors, recent work has explored three main sensing modalities: the electrocardiogram (ECG), seismocardiogram (SCG), and photoplethysmogram (PPG). Characterizing the conduction of cardiac action potentials, the ECG has been extensively used to glean insight into autonomic control of the heart via analysis of HR variability (HRV) (*5*). In contrast, the SCG is a mechanical signal that measures the movement of the chest wall in response to underlying cardiovascular events. Prior work has used SCG, which has been deployed on numerous wearable systems (*6*, *7*), to estimate pre-ejection period (PEP) and left ventricular ejection time (LVET), two important features of cardiomechanical function (*8*). Last, the PPG is an optical signal that is used to monitor the flow of blood through peripheral vasculature (*9*). A common feature derived from PPG is the pulse transit time (PTT)—or the delay between ejection of blood from the heart and its arrival at a distal location—which is often used to estimate BP (*10*). Other amplitude and morphological features of the PPG such as the pleth variability index (PVI) have been related to various outcomes, including fluid responsiveness and vascular tone (*11*, *12*). Together, these three sensing modalities provide a holistic electromechanical view of cardiac function and its interaction with the broader vascular system; for this reason, the wearable approach to BVS estimation used in this work fused all three of these sensing modalities.

A catheter-based system was used in this study to provide a baseline of comparison for the wearable system. This was performed because catheter-based systems allow for the direct measurement of vascular pressure gradients during the cardiac cycle. Because of their invasive nature, these systems are not typically used outside the surgical setting; however, this allowed for the direct measurement of cardiac time intervals and vascular pressures to serve as baseline references in this study. With this system, pressures were recorded in four different locations: the aortic root, femoral artery, right atrium, and left atrium. As will be described, aortic root and femoral artery pressures may be used in conjunction to obtain references for the SCG- and PPG-derived cardiomechanical and vascular features. Furthermore, right and left atrial pressures—the latter of which is estimated via pulmonary capillary wedge pressure (PCWP)—are both classically used as analogs of preload, with the former reflecting venous return and the latter being modulated by left ventricular pressure (*13*, *14*). In addition to obtaining reference values for the physiological features from the wearable system, vascular pressure features obtained from the catheter-based system included mean aortic root pressure (MARP), mean femoral artery pressure, mean PCWP, and mean right atrial pressure. Last, pulse pressure variability (PPV) was assessed from the femoral artery pressure waveform, serving as an analog for PVI obtained from the wearable system.

An experimental protocol was conducted on six pigs (weight, 51.5 to 71.4 kg) according to a protocol approved by the Institutional Animal Care and Use Committees of the Georgia Institute of Technology, Translational Testing and Training Labs Inc. and the Department of the Navy Bureau of Medicine and Surgery. In this study, hypovolemia was induced by draining blood through an arterial line at four levels of blood volume loss (7, 14, 21, and 28% of total blood volume), after each of which exsanguination was paused for approximately 5 to 10 min to allow the cardiovascular system to stabilize. If cardiovascular collapse occurred once a level was reached, as defined by a 20% drop in MARP from baseline after stabilization, then exsanguination was terminated. Notably, all animals reached this 20% cutoff during the protocol, allowing it to serve as a reference for cardiovascular collapse across all animals.

### Comparison of feature quality from wearable- and catheter-based systems

Figure 1C shows example traces of wearable- and catheter-based modalities over a single respiration cycle for one of the animals at baseline. From the traces shown in Fig. 1C, a number of physiological features were derived; these are summarized in Table 1, and a detailed description of the etiology and calculation of each is provided in the Supplementary Methods. Because some features may be specific to catheter-based or wearable systems, Table 1 also specifies the sensing system from which each feature was derived. For each signal, the R-peaks of the concurrent ECG waveform were used to separate the signals on a heartbeat-by-heartbeat basis; hence, a single estimate for each physiological feature used in this study was obtained for each heartbeat.

**Table 1:** List of physiological features and their corresponding sensing system.

**Feature name**	**Sensing system**
Heart rate (HR)	Both
Time domain heart rate variability (HRV)	Both
Frequency domain (spectral) HRV	Both
Poincaré plot (dynamic) HRV	Both
Pre-ejection period (PEP)	Both
Left ventricular ejection time (LVET)	Both
PEP-to-LVET ratio (PEP/LVET)	Both
Pulse arrival time (PAT)	Both
Pulse transit time (PTT)	Both
HR-normalized PAT (nPAT)	Both
Pleth variability index (PVI)	Wearable
Pulse pressure variability (PPV)	Catheter
Mean aortic root pressure (MARP)	Catheter
Mean femoral pressure (MFP)	Catheter
Mean PCWP (MPCWP)	Catheter
Mean right atrial pressure (MRAP)	Catheter

**Fig. 2:**
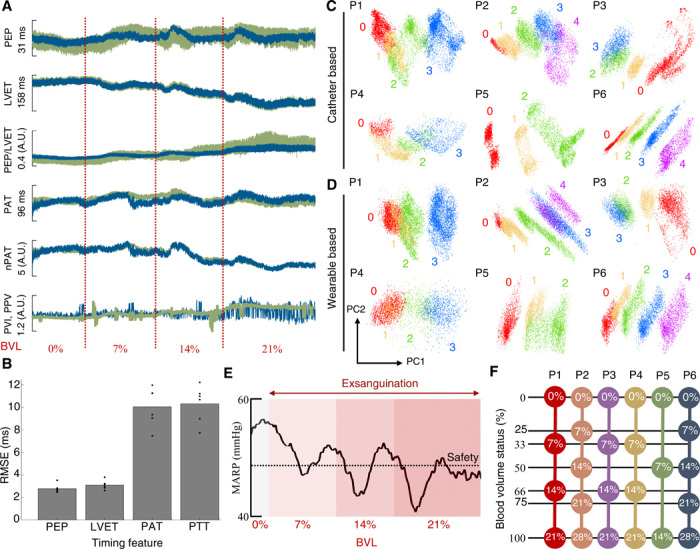
Physiological features during exsanguination. (**A**) Example features derived from catheter-based (green) and wearable (blue) sensors during the experimental protocol for pig 1. All features were mean centered over the recording session for visualization. Features are plotted against blood volume loss (BVL), with the red dotted lines indicating the onset of each blood draw. nPAT, normalized PAT. (**B**) Error of timing features calculated from wearable-based signals with respect to the catheter-based reference. Overlaid scatter points represent individual animals. (**C**) Results of performing principal components analysis (PCA) on all catheter-based features from Table 1 over the recording session for pigs 1 to 6, respectively. Number labels represent the BVL corresponding to each data point: 0% (0), 7% (1), 14% (2), 21% (3), and 28% (4). (**D**) Results of PCA on all wearable-based features from Table 1 for pigs 1 to 6, respectively. (**E**) Heartbeat-by-heartbeat MAP for pig 1 over the experimental protocol. Progressive BVL is indicated by red shading, and the 20% safety threshold is overlaid (black, dotted). (**F**) Graphical representation of BVS calculation from raw BVL for each animal. PN, pig N; RMSE, root mean square error; A.U., arbitrary units. Detailed explanation is provided in the text.

Figure 2 (A to D) demonstrates the correspondence between features derived from the catheter-based and wearable systems. Figure 2A provides an example of the features from Table 1, which are computed separately from the two sensing systems for one of the animals during exsanguination. As illustrated, wearable-based features closely corresponded with their catheter-based counterparts except for PVI, which was substantially more prone to noise than catheter-derived PPV. This is likely due to noted limitations of PPG signals, the amplitude of which is subject to a variety of confounding factors and artifactual noise (*9*). Figure 2B shows the error between the timing features from Fig. 2A derived from wearable sensors and their catheter-based counterparts. This figure shows that PEP, LVET, pulse arrival time (PAT), and PTT estimation were within acceptable limits based on prior studies in the wearable-based estimation of these features (*15*, *16*). Notably, features for which acceptable error limits have not been postulated or which derive from these features were excluded from this figure; however, figs. S1 to S3 provide a detailed overview of the trends in each feature used in this study.

Figure 2 (C and D) shows the results of performing principal components analysis (PCA) on the physiological features of each animal during the experimental protocol. As illustrated in these figures, a consistent, sequential progression through blood volume levels is apparent using both the catheter-based and wearable systems; both sets not only achieve consistent separation between the different blood volume levels but also share morphological similarity for the same animal across the two systems. While these results do not suggest that generalized BVS assessment is possible, the consistent stratification of blood volume levels achieved by data from the two sensing systems qualitatively demonstrates that (i) both systems were able to capture physiological changes occurring during exsanguination and (ii) both systems were similarly affected by these changes. This suggests that, from a qualitative standpoint, data from the wearable system had comparable integrity to that of the catheter-based system for this task.

### Defining BVS

As aforementioned, the experimental protocol was terminated for each animal once MARP remained below the predefined safety threshold after allowing several minutes for cardiovascular compensation following each exsanguination step. Figure 2E shows the MARP over the experimental protocol for pig 1; as illustrated, MARP remained below the safety threshold after 21% of the total blood volume was extracted. For the six animals in the study, this safety threshold was reached after differing levels of blood volume loss: pigs 1, 3, and 4 reached 21% BVL; pigs 2 and 6 reached 28%; and pig 5 reached only 14%.

For this reason, it is important to know a patient’s progression toward cardiovascular collapse rather than raw blood volume loss for the purposes of triage. To model this progression, percent BVL was converted to BVS for each animal, as detailed in Fig. 2F. Specifically, BVS was calculated via

$$\text{BVS}=\frac{\%\text{BVL}}{\%{\text{BVL}}_{\text{max}}}$$ (1)

where %BVL_max_ is the percent blood volume loss at which the safety threshold was reached. In this manner, BVS was expressed on a scale from 0 (0%) at baseline to 1 (100%) at the safety threshold. As shown in Fig. 2F, BVL was thereby adjusted by each animal’s tolerance to exsanguination to obtain a more consistent metric for comparison. This definition of BVS is analogous to the compensatory reserve index (CRI) described in (*17*). Specifically, BVS is related to the CRI via CRI = 1 − BVS.

### BVS estimation using ensemble regression

To develop a globalized model of BVS assessment, random forest regression was used to learn a mapping between the observed physiological features and BVS. Random forest models are a type of ensemble learner composed of numerous regression trees, each of which estimates the outcome of interest based on the input features (*18*). Random forest regression was chosen because of its ability to model highly nonlinear relationships between features while also providing insight into their relative importance for BVS assessment (*19*).

**Fig. 3:**
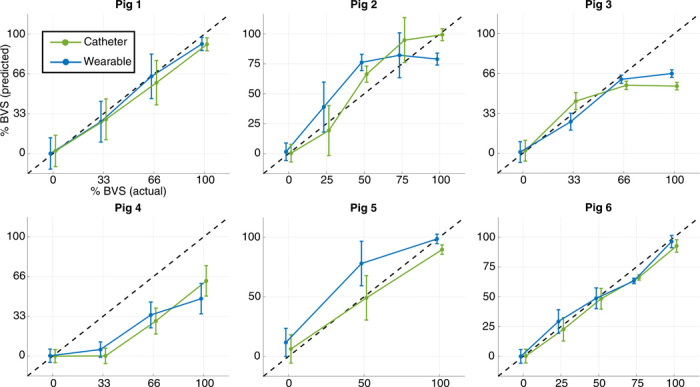
BVS prediction results. Results of performing random forest regression with cross-validation to predict BVS for each of the animals. Separate models were trained for this task using the catheter-based (green) and wearable (blue) systems. SD bars are shown for predicted BVS at each level; the 1:1 correspondence line between actual and predicted BVS is overlaid (black, dashed).

Separate random forest models were trained using the leave-one-subject-out cross-validation for the wearable- and catheter-based systems. Hence, two models were tested per animal; the model trained from the wearable feature set used 11 total features, while the model trained using the catheter-based feature set used 15 total features as per Table 1. The trained models were then tested on the data from the held-out animal. The error between the true and estimated BVS was then recorded to assess performance. The outcome of this analysis is shown in Fig. 3, in which the mean and SD of predicted BVS using both systems were plotted at each blood volume level and for each animal subject. Of note in the result is the similarity in performance between the wearable- and catheter-based systems for this task. Except for the initial level of BVL for pig 4 and the final level for pig 3, which will be discussed in the following section, there was a strong qualitative association between predicted and true BVS for both systems.

### Analysis of physiological features during exsanguination

**Fig. 4:**
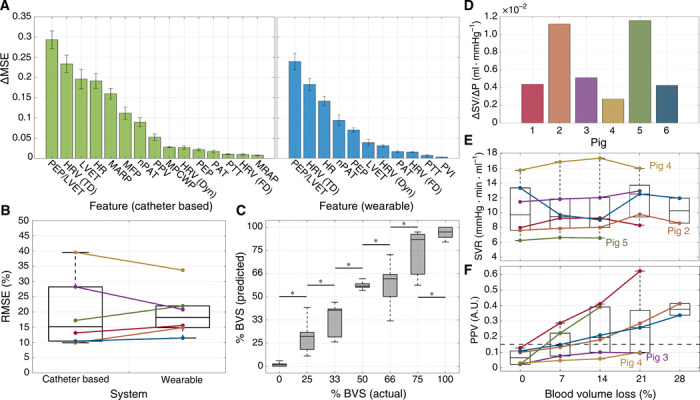
Analysis of physiological features during exsanguination. (**A**) Feature importance for catheter-based (green) and wearable (blue) systems during random forest regression. SD error bars are shown. (**B**) RMSE between ground truth and estimated BVS using random forest regression (from Fig. 3). Colors for all figures correspond to the labeling in (D). (**C**) Actual versus predicted BVS aggregated across all animal subjects for the wearable system. Boxes denote the 25th to 75th percentile range, and whiskers denote the 5th to 95th percentiles. Asterisks indicate significant separation (*P* < 0.05) between the levels as determined by a Mann-Whitney *U* test. (**D**) Slope of linear fit between stroke volume (SV) and preload. (**E**) Average systemic vascular resistance (SVR) computed at each level of BVL. (**F**) Average PPV computed at each level of BVL. A reference line at 0.15 is overlaid (black, dashed). MSE, mean square error.

Figure 4A shows the resulting importance calculated for each physiological feature in the study. For both systems, the ratio of PEP to LVET was the most important physiological feature for BVS estimation, far exceeding the traditional features HR and MARP for this task. As apparent in fig. S1 to S3, most features studied in this work had heterogeneous responses to exsanguination, making it difficult to infer BVS from these biomarkers. An example is shown in fig. S2A; while HR would typically be expected to increase during exsanguination, it paradoxically decreased in two of the six animal subjects in this study. In contrast, the response of PEP/LVET (PEP-to-LVET ratio) to exsanguination was not only consistent across animal subjects, but appreciable separation was present between blood volume levels, as illustrated in fig. S1. A possible explanation for this result is that PEP/LVET has been found to strongly predict changes in ejection fraction—and thereby, left ventricular function—which undergoes marked changes during hypovolemia (*20*, *21*).

Of note is that PEP/LVET was substantially more useful for BVS estimation than either feature in isolation. Although PEP was not consistently affected by exsanguination compared with LVET (fig. S1), this information was still essential in modulating the more consistent LVET to produce the most reliable feature for this task. This result illustrates that lack of a consistent trend in a feature does not necessarily indicate that it is devoid of useful information. Of further note is that cardiomechanical features were shown in Fig. 4A to outperform arterial and venous pressures for this task. Ultimately, the results of Fig. 4A indicate that capturing cardiomechanical signals with wearable monitoring systems may hold the key to enabling noninvasive, real-time monitoring of BVS.

These results also corroborate the findings of prior work regarding the importance of HRV in assessing BVS. Among the tested features, time-domain HRV was a robust predictor of BVS, far outpacing both frequency-domain analysis and Poincaré plots. While trends in frequency-domain HRV reflect the expected increase in sympathetic tone during exsanguination, these latter two methods were inconsistent and prone to outliers, which made it difficult to infer BVS using these features (fig. S2). Even so, it should be noted that time-domain methods of HRV estimation may correlate strongly with HR itself; this is shown quantitatively in fig. S4, which reports the coefficient of determination between each pair of features in this study. The strong correlation between time-domain HRV and HR suggests that this method may be prone to the same pitfalls of using HR despite its usefulness for BVS estimation, that it is not specific to changes in blood volume and should be subject to additional scrutiny.

On the basis of Fig. 4A, features based on pulse wave propagation times performed relatively poorly for BVS estimation. Notably, prior research has suggested that pulse wave velocity in the vascular system is a complex process governed by several factors independent of BP itself (*9*, *10*). As a result, PAT and PTT are often related to BP via a linear fit that is both subject specific and transient due to varying physiological state (*10*). During the experimental protocol, neither PAT nor PTT exhibited consistent trends, resulting in poor performance for this task (fig. S1). While HR-normalized PAT exhibited stronger performance, this is likely due to the strong correlation between HR-normalized PAT and HR rather than inherent novelty of this feature (fig. S4). In contrast to PAT and PTT, PVI and PPV experienced a general increase during exsanguination; however, in a similar manner, strong consistency was not exhibited between animal subjects (fig. S1). As a result, Fig. 4A shows that PVI performed poorly for BVS estimation as well.

### Performance of BVS estimation

Regarding the performance of the wearable system for this task, Figs. 3 and 4B illustrate the similarity in performance between the wearable system and the catheter-based gold standard. These findings, combined with the unsupervised results of Fig. 2 (C and D), suggest that it is not only possible to determine the severity of exsanguination completely noninvasively, but that this may be accomplished with accuracy comparable to invasive reference standards.

To compare the performance of the proposed model to prior literature in this field, the regression problem in this work was reframed as a classification problem. Specifically, the predicted BVS from the catheter-based and wearable systems from Fig. 3 was used to classify each heartbeat from all six animal subjects as occurring before (BVS < 1) or after (BVS = 1) cardiovascular collapse. This process was then repeated using each of the individual physiological features used in this study. The results of this analysis are shown in table S1. Most notably, the catheter-based system achieved a sensitivity, specificity, and area under the receiving operator characteristic (ROC) curves of 0.84, 0.95, and 0.93, respectively; the wearable system achieved values of 0.75, 0.90, and 0.91 respectively. To place these values in context, comparable performance was reported by Hinojosa-Laborde *et al.* in (*22*), which performed a similar study in nonhuman primates, and Howard *et al.* in (*17*), which used the same method as in (*22*) to detect cardiovascular collapse in human subjects undergoing lower-body negative pressure.

As the proposed wearable system and associated models would be a novel addition to the clinical environment, it is difficult to translate the implications of the reported error and performance metrics to the context of trauma care. To provide this perspective, Fig. 4C investigates the efficacy of the proposed model based on the wearable system as a digital biomarker of hemorrhage severity. In the figure, the data presented in Fig. 3 were aggregated across all animal subjects to observe the relationship between true and predicted BVS at each level. To determine whether significant differences (*P* < 0.05) were present in predicted BVS between adjacent levels, a Mann-Whitney *U* test was performed between the data in each pair of adjacent levels. The results of Fig. 4C illustrate not only a strong relationship between true and predicted BVS from the wearable system but also that the change in predicted BVS was significant at each level.

### Influence of physiological state on BVS estimation

As aforementioned, the animals’ physiological set points at the beginning of the experiment may have had substantial impacts on the results shown in Fig. 3. To assess these differences, Fig. 4 illustrates several important markers of cardiovascular function. Figure 4D shows the change in stroke volume (SV) versus the change in preload during the experimental protocol. Compared with the other animals, pig 4 had a substantially muted SV response to changes in preload, a paradoxically decreasing HR throughout the experiment, as shown in fig. S2A, and a consistently high systemic vascular resistance (SVR), as shown in Fig. 4E. Because of this, pig 4 appeared to have a relatively dampened physiological response to hypovolemia. This observation is notable in the context of Fig. 3, which shows that the blood volume loss of pig 4 was severely underestimated due to the lack of an increase in predicted BVS during the first blood draw.

Figure 4F provides more context for this observation. PPV is often used to determine whether a patient is a “fluid responder,” meaning that his or her cardiac output (CO) is expected to have a large response to fluid resuscitation (or, conversely, hemorrhage). In contrast, patients whose CO has a more modest response to fluid resuscitation is called a “fluid nonresponder.” One way to determine fluid responsiveness is already shown in Fig. 4D, which shows that pig 4 had a modest SV response to changes in preload. In this case, SV was used as a proxy for CO. Another way to assess fluid responsiveness is using PPV; it is commonly noted in the literature that patients with a PPV under 15% (0.15) may be classified as fluid nonresponders (*23*). As shown in Fig. 4F, pigs 3 and 4—whose BVS was underestimated by the model—may be classified as fluid nonresponders. Typically, this phenomenon is understood in terms of the Frank-Starling curve, with fluid responders falling on the high-slope region of the curve and nonresponders falling on the low-slope region of the curve.

The difference in physiological state for pig 4 may have been due to the modification of the experimental protocol for this animal, as detailed in Materials and Methods. The administration of atropine to compensate for low starting BP led to a baseline HR that was already elevated; coupled with the results in Fig. 4 (D to F), it is likely that pig 4 was already compensating for hypotension when the experiment began, and therefore, the additional perturbation of induced hypovolemia did not elicit a strong response in this animal. In contrast, Fig. 4 (D and E) shows that pigs 2 and 5 had a large SV response to changes in preload coupled with a relatively low SVR, suggesting that these animals had a larger cardiovascular response to blood volume changes. Figure 3 shows that the blood volume loss of pigs 2 and 5 was somewhat overestimated.

## DISCUSSION

The findings in Fig. 4A that the cardiomechanical feature PEP/LVET was an essential physiological feature for estimating BVS is critical in the context of cardiac monitoring systems. Although the heart is a complex organ with both electrical and mechanical function, wearable cardiac monitoring systems have traditionally focused on quantifying the heart’s electrical activity via ECG in lieu of its mechanical function. However, recent advances in wearable sensing and machine learning have enabled the noninvasive assessment of cardiomechanical function. As apparent from Fig. 4A, assessing the mechanical function of the heart via modalities such as SCG may hold the key to achieving accurate triage and management of trauma injuries via wearable systems. More broadly, the results of Fig. 4C suggest that the proposed model deployed on a multimodal wearable system was an effective digital biomarker of hemorrhage severity in this cohort of animal subjects; while this model may not translate directly to human patients, these findings represent an interesting predicate that warrants further exploration in larger cohorts.

The results of Fig. 3 further illustrate the role of BVS estimation in the triage of absolute hypovolemia. Consider the subplots for pigs 5 and 6 in this figure; although pig 6 did not reach the safety threshold until 28% of its blood volume was extracted, pig 5 reached the threshold after only 14% BVL. A system that simply reported BVL would therefore reflect the same severity of absolute hypovolemia for pigs 5 and 6, with a 14% loss in blood volume. Not only is this task more difficult due to the animal-specific response to hypovolemia—which depends heavily on the initial physiological state—but it is also less useful in the context of triage. With the exception of pig 4, which underwent complications during its experimental protocol, the results in Fig. 3 reflect the relative severity of given levels of BVL across the animals in terms of their progression toward cardiovascular collapse.

As was explored in Fig. 4, heterogeneous responses to hypovolemia and variable physiological set points at its onset remain important challenges to overcome for the accurate assessment of hypovolemia, whether via wearable systems or otherwise. For this reason, future work should investigate how the physiological set point of each animal can be assessed with wearable systems and integrated into predictive models of BVS. The dependence of model performance on the animals’ prehypovolemic physiological set point is an important avenue for future work. To further enable more robust and generalizable BVS estimation, studies should explore how these varying set points may be quantified with wearable sensing and used to modulate estimates of BVS. More broadly, it is likely that the proposed model does not account for physiological mechanisms affected by progressive hypovolemia, a limitation that should be explored in future work to improve performance. Even so, the observation that high performance was possible for most animals using the proposed model, especially in the context of held-out cross-validation applied to a relatively small dataset, suggests that the proposed model may become more robust and generalizable with larger, diverse training sets.

It is notable that a static regression model—random forest regression—was used rather than time series models such as hidden Markov models or recurrent neural networks. This was done because the experimental protocol in this work was not representative of a natural process of exsanguination, as blood loss through a venous line was interspersed with pauses for cardiovascular compensation. Time series models may be more suitable for this task, as they are able to assess longer-term physiological changes; however, this necessitates that temporal patterns used to train the models be reflective of the use-case of the technology, a challenging feat for human and animal subjects alike. Although the aims of this work were still achievable with random forest regression, future studies may seek to improve performance by exploring various time series modeling approaches.

The proposed model for detecting cardiovascular collapse may have crucial implications for the eventual deployment of these systems, especially in the context of prior work such as the PPG-based compensatory reserve. Specifically, developing systems that fuse both SCG- and PPG-based methods may improve the translation of this research to trauma settings, perhaps via a synergistic effect between the modalities, by adding redundancy in the system and by examining both central factors (the pump) and peripheral factors (the pipes). As an example, in trauma settings involving burns and amputations, it may be more feasible to rely on either SCG or PPG, depending on the nature of the injury; furthermore, if either wearable modality returns low-quality data, then redundancy in the system may still allow robust inference of BVS.

In the near future, assessing cardiomechanical function using seismocardiography may enable noninvasive, real-time management of hemorrhage-induced hypovolemia in clinical settings. In practice, ECG and SCG sensors may be implemented on small wireless patches such as those previously developed for heart failure monitoring (*7*); furthermore, PPG sensors may be miniaturized and packaged for clinical deployment as well, as is already done with pulse oximetry. While wearable systems for the deployment of these modalities have already been developed, recent advances in noncontact sensing may soon enable the assessment of ECG- (*24*), SCG- (*25*), and PPG-derived physiological features (*26*) in scenarios where bodily trauma prevents the placement of these wearable sensors.

Ultimately, validating the clinical results of this work and the proposed BVS assessment will require performing this inquiry with human subjects. While the porcine cardiovascular system bears high resemblance to that of humans in many ways, there still exist important anatomical and physiological differences between pigs and humans. As a notable example, it was found in (*27*) that pigs have a critical delivery of oxygen of approximately 11.8 ml O_2_/kg per minute, which is substantially higher than the range found in humans, having been reported as less than 7.3 ml O_2_/kg per minute (*28*). This would indicate that pigs would reach cardiovascular collapse before humans. For this reason and others, the model developed here may not generalize to humans with the current parameterization; rather, the goal of these results is to form a basis for future studies to explore externally measurable changes in cardiomechanical function in humans and, subsequently, to develop a parameterization of the proposed numerical model that achieves high performance in human subjects.

## MATERIALS AND METHODS

### Study design

This protocol included six Yorkshire swine (three castrated male and three female; age, 114 to 150 days; weight, 51.5 to 71.4 kg), each of which passed a health assessment examination but were not subject to other exclusion criteria. Anesthesia was induced in the animal with xylazine and telazol and maintained with inhaled isoflurane during mechanical ventilation. Intravenous heparin was administered as needed to prevent coagulation of blood during the protocol. Before the induction of hypovolemia, a blood sample was taken to assess baseline plasma absorption. Following this baseline sample, Evans Blue dye was administered for blood volume estimation (*29*). After waiting several minutes to allow for even distribution of the dye, a second blood sample was taken to measure plasma volume. In this method, plasma volume is used along with hematocrit to estimate total blood volume. For one animal in the protocol (pig 4), atropine was administered to raise the starting HR and BP due to critically low values.

Hypovolemia was induced by draining blood through an arterial line at four levels of blood volume loss (7, 14, 21, and 28%), as determined by the estimated total blood volume from the Evans Blue dye protocol (*30*). After draining passively through the arterial line, the blood was stored in a sterile container. Following each level of blood loss, exsanguination was paused for approximately 5 to 10 min to allow the cardiovascular system to stabilize. During this time, CO was estimated via thermodilution using a thermistor-enabled Swan-Ganz catheter (*31*). If cardiovascular collapse occurred once a level was reached, as defined by a 20% drop in MARP from baseline after stabilization, then exsanguination was terminated.

Signals from wearable sensors were continuously recorded using a BIOPAC MP160 data acquisition system (BIOPAC Systems Inc., Goleta, California, USA) with a sampling frequency of 2 kHz. As shown in Fig. 1B, ECG signals were captured using a three-lead system of adhesive-backed Ag/AgCl electrodes placed in an Einthoven Lead II configuration, which interfaced with a BIOPAC ECG100C amplifier. Reflectance-mode PPG was captured with a BIOPAC TSD270A transreflectance transducer, which interfaced with a BIOPAC OXY200 veterinary pulse oximeter. The transducer was placed over the femoral artery on either the right or left caudal limb, contralateral to inducer placement. SCG signals were captured using an ADXL354 accelerometer (Analog Devices Inc., Norwood, Massachusetts, USA) placed on the midsternum, interfacing with a BIOPAC HLT100C transducer interface module.

Aortic root pressure was captured by inserting a fluid-filled catheter through a vascular introducer in the right carotid artery, fed through to the aortic root. Femoral artery pressure was obtained directly from an introducer placed on either the left or the right femoral artery depending on accessibility. Right and left atrial pressures were captured with a Swan-Ganz catheter with proximal and distal monitoring ports inserted in either the right or the left femoral vein. This setup is illustrated in Fig. 1B. Left atrial pressure was inferred via PCWP captured using an Edwards 131F7 Swan-Ganz catheter (Edwards Lifesciences Corp, Irvine, California, USA), which contains a thermistor for performing thermodilution to measure CO. The vascular introducers were connected via pressure-monitoring lines to ADInstruments MLT0670 pressure transducers (ADInstruments Inc., Colorado Springs, Colorado, USA). Data from the catheters were continuously recorded with an ADInstruments Powerlab 8/35 acquisition system sampling at 2 kHz.

### Signal preprocessing

All signals were filtered with finite impulse response band-pass filters with Kaiser window, both in the forward and reverse directions to offset phase shift. Cutoff frequencies were 0.5 to 40 Hz for ECG and 1 to 40 Hz for SCG (*32*). Only the dorsoventral component of the SCG acceleration signal was used in this study. PPG signals, along with all four catheter-based pressure signals, were filtered with cutoffs at 0.5 to 10 Hz (*10*). After filtering, data from all signals were heartbeat separated using ECG R-peaks. The signal segments were then abbreviated to a length of 1000 samples (500 ms) to enable more uniform analysis, which was selected on the basis of the shortest R-R interval in the protocol.

### Outlier identification and removal

The values computed for each physiological feature for each heartbeat during the protocol were processed by a rolling window with a length of 500 heartbeats with 50% overlap between windows. For each window, the mean and SD of the feature were calculated; values that deviated from the mean by greater than 2 SDs were removed. These outliers were then replaced by linearly interpolating their nearest nonoutlier neighbors on either side.

### Principal components analysis

For each animal, all catheter- and wearable-based features were estimated for each of the *N* heartbeats in the experiment, resulting in the column matrices **X**_**C**_∈ℝ^*N* × 15^ and **X**_**W**_∈ℝ^*N* × 11^ based on the distribution of features described in Table 1. PCA was performed on each of these matrices individually, and the first two principal components resulting from the analysis are plotted in Fig. 2 (C and D).

### Formatting data for model training

For each animal, the calculated features were expressed as their percent change from baseline rather than their raw values. This was performed to account for the different baseline set points of each feature, focusing instead on changes in the feature. To do so, for each animal, the *i*th feature **x***_i_* ∈ **X**, **X** = [**X**_**C**_, **X**_**W**_] was recalculated via

$$x_{i}^{\prime }=\frac{x_{i}-x_{i}^{\left(0\right)}}{x_{i}^{\left(0\right)}}$$ (2)

where $x_{i}^{\left(0\right)}$ is the initial value of the *i*th feature. The updated values $x_{i}^{\prime }$ were then used for further analysis.

### Random forest training and testing

In this work, random forest classifiers were trained using bagging to increase the diversity of the ensemble while decreasing the risk of overfitting (*33*). To train a random forest in this manner, each regression tree in the ensemble was trained using a randomized subset of the samples. The output of the ensemble was taken as a simple average across individual regression trees, with the performance of the model assessed using the mean square error between the estimated and true BVS.

When training a random forest model using bagging, important hyperparameters include the number of regression trees in the ensemble and the maximum depth—or number of consecutive splits—of each tree. Increasing the former in turn increases the diversity of the ensemble; however, it increases training time and complexity of the model. Increasing the latter may result in improved accuracy, although excessively deep trees may overfit the training data (*34*). Because of this, a hyperparameter sweep was performed over a range of ensemble sizes and tree depths to analyze the effect of each parameter on performance. An additional notable hyperparameter for these models is the maximum number of features sampled at each node, since sampling a subset of features for each learner and again at each node may increase the diversity of trees within the ensemble (*35*). However, to obtain an unbiased view of relative feature importance, and because of the small size of the feature set, all features were equally likely to be sampled at each node in this work, and the number of features that could be sampled at each node was not limited.

Figure S5 shows the results of performing this hyperparameter sweep on the data using cross-validation. Of note in this figure is that tree depth has a greater influence on overall performance than the size of the ensemble. Large changes in prediction error are apparent for low depths; however, predictive performance begins to level off rapidly as depth is increased. In contrast, performance changes due to ensemble size are negligible beyond approximately 20 regression trees. Notably, once tree depth is sufficient, the prediction error is relatively stable for small changes in the hyperparameters; this indicates robustness of the model, as hyperparameter selection did not have a substantial impact on the results. On the basis of the results of this sweep, an ensemble of 100 trees with depth of 10 was used for the analysis in this work.

Training and testing were performed using the MATLAB regression learner package. To perform cross-validation, six separate models were trained for both the catheter- and wearable-based feature sets, each leaving out data from one of the six animal subjects during training. The trained model generated by the MATLAB package was then tested using the data from the held-out animal subject.

### Feature importance calculation

Feature importance for the wearable- and catheter-based systems was computed by training random forest models on all experimental data in the protocol. The importance of each feature was estimated by permuting its values in the out-of-bag training data for each tree (*18*); the resulting change in mean square error when testing the out-of-bag data across all trees before and after permutation was averaged to estimate the feature importance. Since feature importance may vary based on the random seed used to generate the model, training and feature importance calculations were repeated 100 times with different random seeds for both the wearable- and catheter-based models.

### Computing changes in SV versus changes in preload

To obtain a more complete view of the physiological state of each animal, two quantities were computed using CO: (i) the change in SV versus the change in preload (ΔSV/ΔP) and (ii) SVR. Because afterload and contractility vary during hypovolemia, ΔSV/ΔP does not indicate the slope of the Frank-Starling curve; however, it is still a useful analog of the SV response to preload and was calculated for each animal (*36*). To compute this quantity, SV was calculated from CO via the equation CO = SV × HR. As CO was obtained after each stepwise change in blood volume, SV was therefore computed once per blood volume level per animal over the protocol. ΔSV/ΔP for each animal was then found by computing a line of best fit between SV and preload, as defined by mean right atrial pressure at the time of CO calculation (fig. S6) (*36*, *37*).

### Assessing performance for detecting cardiovascular collapse

Each heartbeat from all animals in the study was labeled with a 0 or 1, with the former indicating a true BVS < 1 and the latter indicating that true BVS = 1 (decompensation). The ability for the wearable- and catheter-based systems to predict whether each heartbeat represented compensation or decompensation was then assessed as follows. A threshold was applied to the estimated BVS for all heartbeats in the protocol computed using the cross-validation procedure. The threshold was swept over the range of BVS values, and the resulting predicted labels were recorded for each value of the threshold; the sensitivity and specificity of the predicted labels for each threshold level were calculated and used to form an ROC curve. The overall sensitivity and specificity were obtained by selecting the point on the ROC curve closest to the upper-left vertex (0, 1). The area under the ROC curve was then computed by integrating the curve in the *x* axis range [0, 1]. This process was then repeated using the values of each feature individually.

## Supplementary Material

abb1708_SM.pdf

## SUPPLEMENTARY MATERIALS

Supplementary material for this article is available at http://advances.sciencemag.org/cgi/content/full/6/30/eabb1708/DC1
