# Supplementary material for: Enabling the assessment of trauma-induced hemorrhage via smart wearable systems
Source: Sci Adv. 2020 Jul 22;6(30):eabb1708. doi: 10.1126/sciadv.abb1708 (PMC7375804; doi:10.1126/sciadv.abb1708)
Supplement: abb1708_SM.pdf [file abb1708_SM.pdf]

advances.sciencemag.org/cgi/content/full/6/30/eabb1708/DC1

Supplementary Materials for

Enabling the assessment of trauma-induced hemorrhage via smart wearable systems

Jonathan Zia*, Jacob Kimball, Christopher Rolfes, Jin-Oh Hahn, Omer T. Inan

*Corresponding author. Email: zia@gatech.edu

Published 22 July 2020, *Sci. Adv.* **6**, eabb1708 (2020)
DOI: 10.1126/sciadv.abb1708

This PDF file includes:

Supplementary Methods
Table S1
Figs. S1 to S7
References

Supplementary Methods

Several criteria were used to select the features assessed in this work. Most importantly, features were selected that have been shown in previous studies to correlate with changes in blood volume while also having a clear physiological etiology; thus, feature-mining methods were not employed in this study. Though prior work has shown a correspondence between SCG (*38*) and PPG amplitude (*39*) and hemodynamic features related to hypovolemia, obtaining consistent, high-quality amplitude features may be difficult in austere environments. Therefore, physiological features derived from signal amplitude itself were excluded from this work.

i. *Heart Rate Variability (HRV)*

The effect of BVS on HRV has been extensively observed in prior studies (*40, 41*). To compute HRV in this work, a vector of R-R intervals (RRIs) was first created for each recording session by computing the difference between adjacent elements in the vector of ECG R-peak indices (in samples) for the session. This vector was then converted from samples to seconds by dividing by the sampling frequency of 2kHz. For each point in the resulting vector, the HRV was computed by first extracting a five-minute window of RRIs centered on the point, truncated where insufficient data was available at the beginning and end of the session. The HRV for each window was then calculated using various methods.

Notably, HRV may be assessed using a variety of time- and frequency-domain analysis methods, or via Poincaré plots (*42*). Time-domain methods quantify the variability in RRIs over the recording session. This is often performed by simply computing the standard deviation of the

vector of RRIs (*5*). To compute time-domain HRV in this work, each window was first de-trended to remove linear trends in HR before obtaining the standard deviation of the resulting vector.

Frequency-domain methods are based on the observation that sympathetic control of heart rhythm leads to increased low-frequency (LF) content of RRIs, while increased parasympathetic control leads to increased low- and high-frequency (HF) content (*43*). The ratio of LF to HF content is therefore used to infer sympathetic-parasympathetic balance. To compute this value, each window of RRIs was first up-sampled using spline interpolation such that the length was increased to the nearest power of two. The fast Fourier transform algorithm was then applied to the window and the power spectral density was computed as the squared amplitude of the Fourier transform output. An example result is shown in **Supp. Fig. S7**(a). LF power was obtained by integrating the PSD from 0.04Hz to 0.15Hz while HF power was obtained by integrating from 0.15Hz to 0.40Hz (*43*). The LF-to-HF ratio was then obtained by dividing the LF power by HF power for each window.

The third common method for computing HRV utilizes Poincaré plots, which capture RRI dynamics (*5*). To employ this method, we consider the column-vector **x** representing a window of RRIs, and the vector $\hat{x}$, which is an offset version of **x** such that $\hat{x}_i = x_{i-1} \forall \hat{x}_i \in \hat{\mathbf{x}}$. As shown in **Supp. Fig. S7**(b), plotting **x** and $\hat{x}$ on a scatter plot results in a set of points that are contained in an ellipse. The red arrows in the figure represent the dimensions of the ellipse corresponding to the eigenvectors of the covariance matrix of [**x**; $\hat{x}$] belonging to the first two eigenvalues λ_1 and λ_2. In a Poincaré plot, λ_1 reflects the range of RRI values while λ_2 reflects RRI variability between consecutive heartbeats. In this work and others, the ratio λ_2/ λ_1 is therefore used to quantify RRI variability between heartbeats normalized by the range in RRIs for the given window (*5*).

ii. *Pleth Variability Index (PVI) and Pulse Pressure Variability (PPV)*

The Frank-Starling curve describes the relationship between CO and preload, which is dependent on afterload and inotropy. When preload is low, small changes in preload lead to large changes in CO, while CO is relatively stable when preload is large (*36*). In the context of fluid resuscitation of hypovolemic patients, the slope of the Frank-Starling curve is thought to determine a patient's fluid-responsiveness, or the physiological response to the addition of fluid to the vascular system (*44*). For patients undergoing mechanical respiration, small changes in preload are induced over the respiration cycle, which lead to cyclical variability in SV and CO. Prior studies have thereby linked respiratory variability in various markers of cardiac function to fluid responsiveness, often serving as a reflection of preload status (*45*).

In this study, respiratory variability is calculated from femoral PPG and femoral pressure waveforms. When computed using PPG sensors, this is commonly referred to as the pleth variability index (PVI), while this quantity calculated from femoral pressure waveforms is known as pulse pressure variability (PPV) (*46*). For both signals, PVI and PPV, respiratory variability was calculated via

$$\frac{PP_{max} - PP_{min}}{0.5 \times (PP_{max} + PP_{min})} \qquad (1)$$

where PP_{max} and PP_{min} are the maximum ranges of the signal during inspiration and expiration respectively. An illustration of PP_{max} and PP_{min} is shown in **Supp. Fig. S7**(c). This calculation resulted in a single value for the PVI for each respiration cycle, which was upsampled using linear interpolation to obtain an estimate for each heartbeat for the purposes of model training.

The PVI is often related to the concepts of PPV and stroke volume variability (SVV), having been shown to correlate strongly with these values (*46*, *47*). Notably, though amplitude-based features were excluded from this work, the reliance of PVI on relative differences in

amplitude rather than amplitude itself led to its inclusion in this study. Furthermore, it should be noted that PVI may not be effective in spontaneously breathing patients, as it is reliant on mechanically-induced changes in preload (*46*). Therefore, PVI it may be inadvisable to integrate in BVS assessment models in which subjects are not mechanically ventilated.

iii. *Pre-Ejection Period (PEP)*

The PEP is an important feature of cardiomechanical function, quantifying the duration of time between ventricular depolarization and aortic opening (AO) (48). Notably, prior studies have correlated the PEP to blood volume changes (*49*). Using the catheter-based system, PEP was computed as the duration between the ECG R-peak and the point of maximal second derivative of the aortic pressure wave before the global maximum. Derivatives were computed in this work by computing the difference between adjacent elements. An illustration is given in **Supp. Fig. S7**(d). PEP may also be estimated using SCG. A previously-reported automated algorithm (*50*) was used to identify the most consistent peak in the first 250ms of each SCG signal segment, which was then selected as the AO point. While the extracted feature may be offset from true AO, changes in these peaks have been shown to strongly co-vary with true AO (*50*). In the same manner, the duration between the ECG R-peak and this value was used to compute PEP from the wearable system.

iv. *Left Ventricular Ejection Time (LVET)*

The LVET is the duration between AO and aortic closing (AC), and is another important cardiomechanical feature that has been shown to co-vary with blood volume (*51*). With the catheter-based system, AC was identified as the point of minimal second derivative after the global maximum, as illustrated in **Supp. Fig. S7**(d). Using the wearable system, AC may be estimated similarly to AO; the same algorithm (*50*) was used to find the most consistent peak in the final

250ms of the SCG signal segment. With SCG-derived AC as well, the estimated interval may be offset from true AC, however the two values typically co-vary strongly. For both the catheter-based and wearable systems, LVET was calculated as the difference between the AO and AC points. Beyond LVET in isolation, the ratio of PEP to LVET (PEP/LVET) is a feature of noted significance in assessing left ventricular function (*20*), and has been shown previously to correlate with changes in blood volume (*52*). Because of this, PEP/LVET was analyzed in this study, and was calculated by dividing PEP by LVET for each heartbeat.

v. *Pulse Arrival/Transit Time (PAT/PTT)*

Although PTT has been studied in simulated hypovolemia (*49*), it has most often been used for cuffless estimation of arterial BP (*10*). In this work, PTT was found by subtracting PEP from PAT. PAT is defined as the duration between the ECG R-peak and blood pulse arrival at a distal location. This was found for the catheter-based and wearable systems by identifying the maximum second derivative of the femoral pressure waveform and femoral PPG waveform respectively, as illustrated in **Supp. Fig. S7**(d).

vi. *HR-Normalized Pulse Arrival Time (nPAT)*

Prior work has utilized nPAT as a correlate of blood volume changes (*53*). During absolute hypovolemia, nPAT is expected to increase due to decreases in RRI magnitude and increases in PEP due to decreased preload (*53*). Since this feature was initially described while using PPG sensors placed on the distal arm, it is more commonly known as the indexed heart-to-arm time when this sensor setup is used. In this work, nPAT was computed for each heartbeat in the recording session by dividing the catheter- and PPG-derived PAT by the ECG-derived RRI.

vii. *Vascular Pressures*

Though subject to compensatory mechanisms, arterial and venous pressures at various locations in the vascular system have been extensively correlated to changes in blood volume (*54*, *55*). In particular, right atrial pressure and PCWP are widely used as analogues of preload, which decreases during periods of decreased blood volume (*13*, *14*). Because of this, all four pressure waveforms were heartbeat-separated, and the mean of the resulting waveform was recorded for each heartbeat. This resulted in four additional features for the catheter-based system: MARP, mean femoral artery pressure, mean right atrial pressure, and mean PCWP. Though estimation of these features was not available for the wearable system, they were included for the catheter-based system to further establish a gold-standard baseline for comparison.

Supplementary Tables

Table S1. Performance of Physiological Features and Overall Models for Predicting Cardiovascular Collapse.

**Feature**	**Catheter-Based System**			**Wearable System**		
	Sensitivity	Specificity	AUC	Sensitivity	Specificity	AUC
HR	-	-	-	0.89	0.90	0.88
HRV (Time-Domain)	-	-	-	0.70	0.99	0.88
HRV (Spectral)	-	-	-	0.24	0.97	0.65
HRV (Poincaré Plot)	-	-	-	0.46	0.96	0.80
PEP	0.60	0.49	0.56	0.60	0.70	0.72
LVET	0.73	0.85	0.86	0.79	0.82	0.81
PEP/LVET	0.66	0.89	0.89	0.83	0.81	0.87
PAT	0.66	0.55	0.60	0.50	0.57	0.55
PTT	0.53	0.54	0.55	0.54	0.47	0.52
nPAT	0.80	0.88	0.84	0.77	0.91	0.86
PPV, PVI	0.71	0.94	0.91	0.55	0.54	0.55
MARP	0.55	0.89	0.87	-	-	-
MFP	0.54	0.89	0.83	-	-	-
MPCWP	0.54	0.55	0.55	-	-	-
MRAP	0.75	0.61	0.75	-	-	-
**Overall Model**	**0.84**	**0.95**	**0.93**	**0.75**	**0.90**	**0.91**

Supplementary Figures

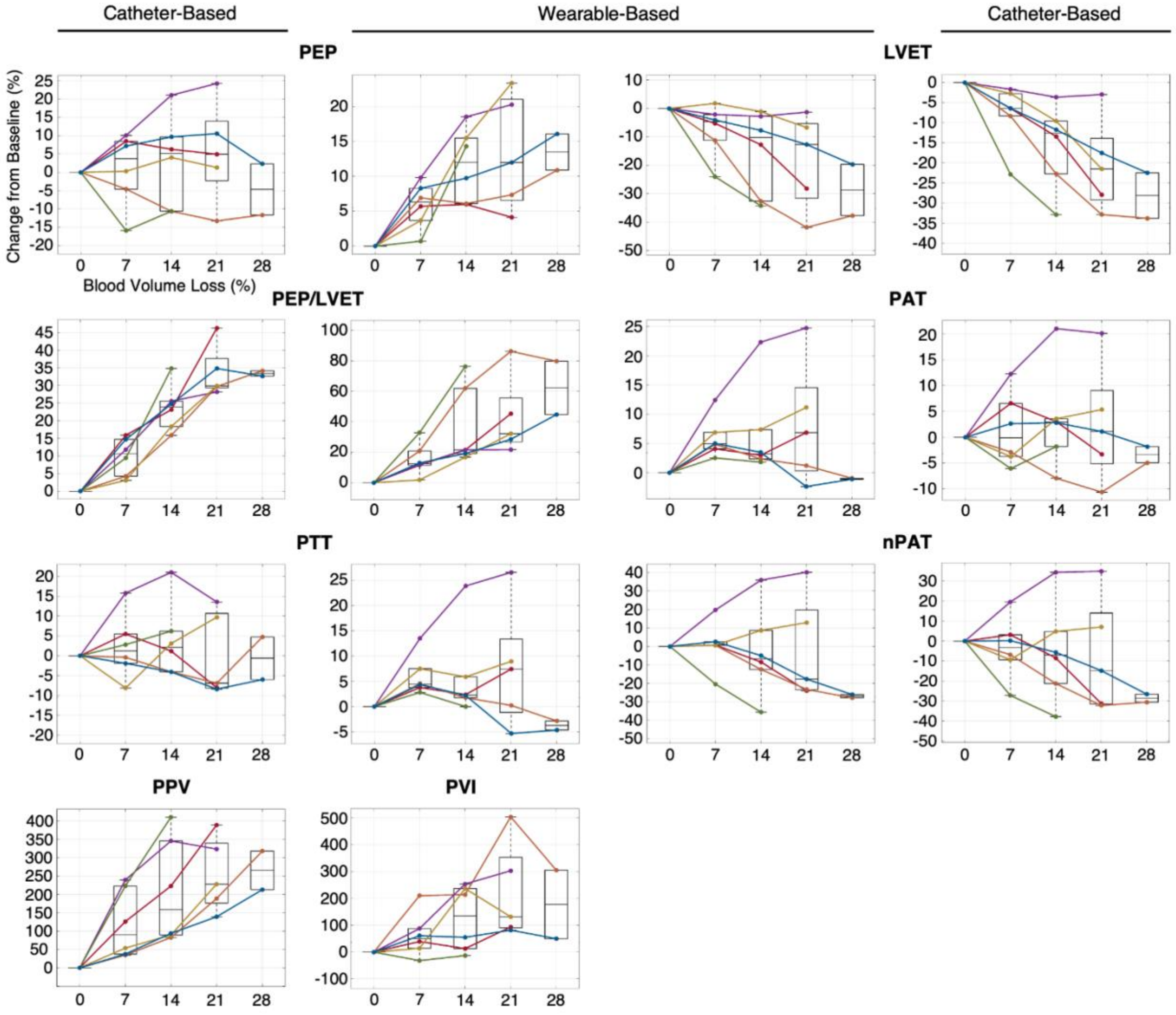

Supp. Fig. S1. Shared Physiological Features during Exsanguination. Average % change from baseline for each level of BVL for each animal. Colors correspond to labels assigned in **Fig. 4(d)**. Features in this figure are those that were computed separately for the catheter-based and wearable systems.

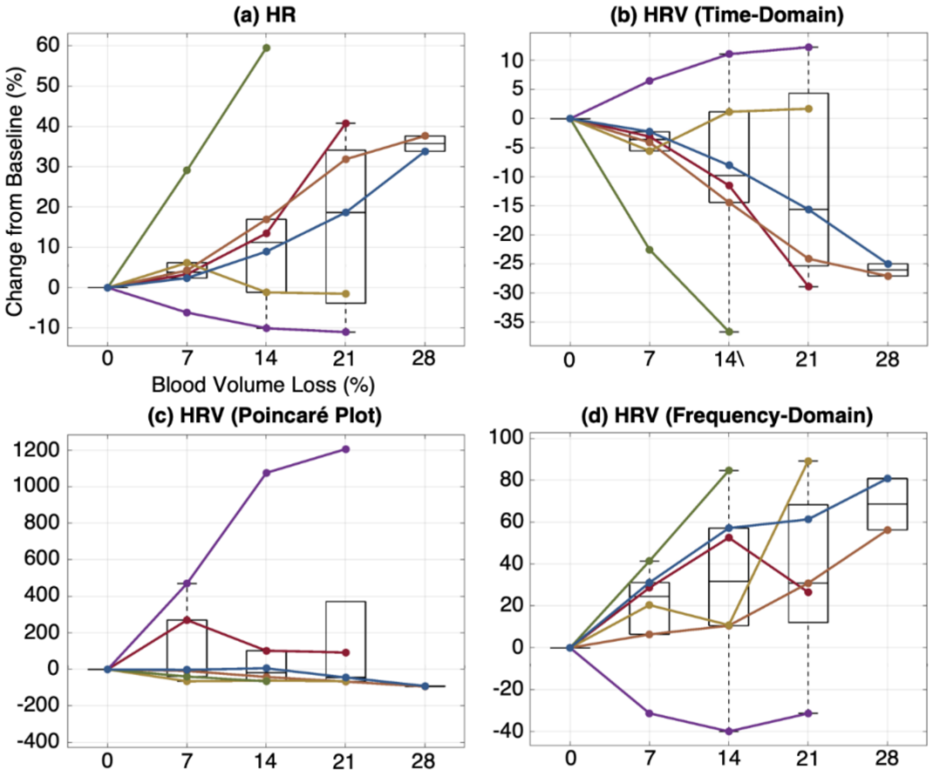

Supp. Fig. S2. HR-Based Physiological Features during Exsanguination. Average % change from baseline for each level of BVL for each animal. Colors correspond to labels assigned in **Fig. 4**(**d**). Features in this figure were derived from the ECG and were included in both the catheter-based and wearable systems.

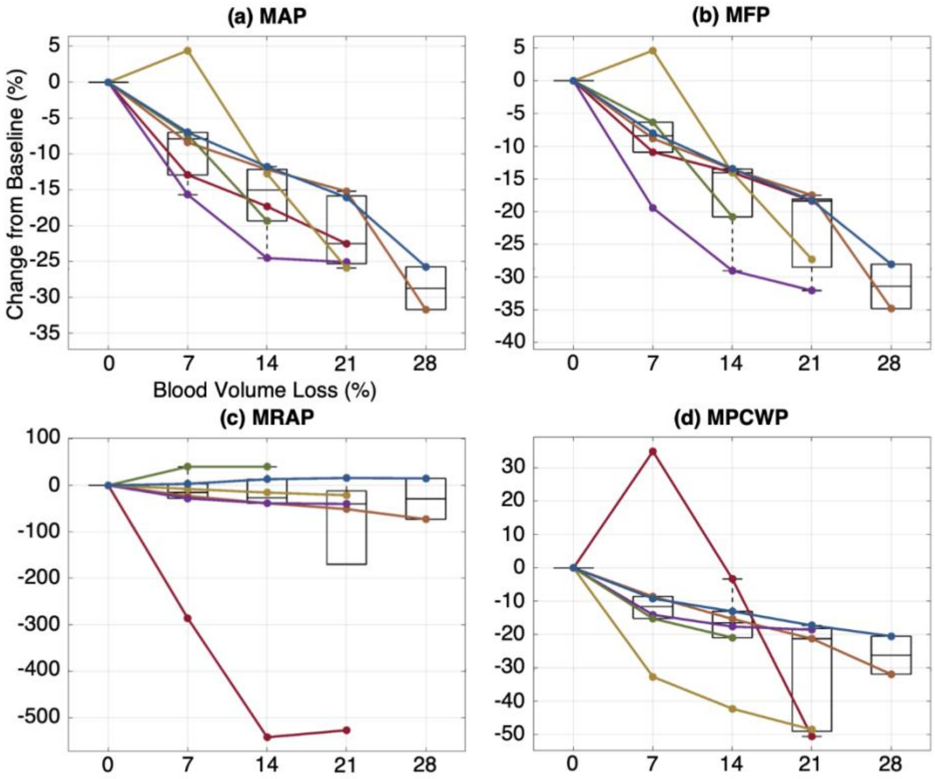

Supp. Fig. S3. Catheter-Based Physiological Features during Exsanguination. Average % change from baseline for each level of BVL for each animal. Colors correspond to labels assigned in **Fig. 4**(**d**). Features in this figure were derived from the catheter-based system only and did not have analogs in the wearable system.

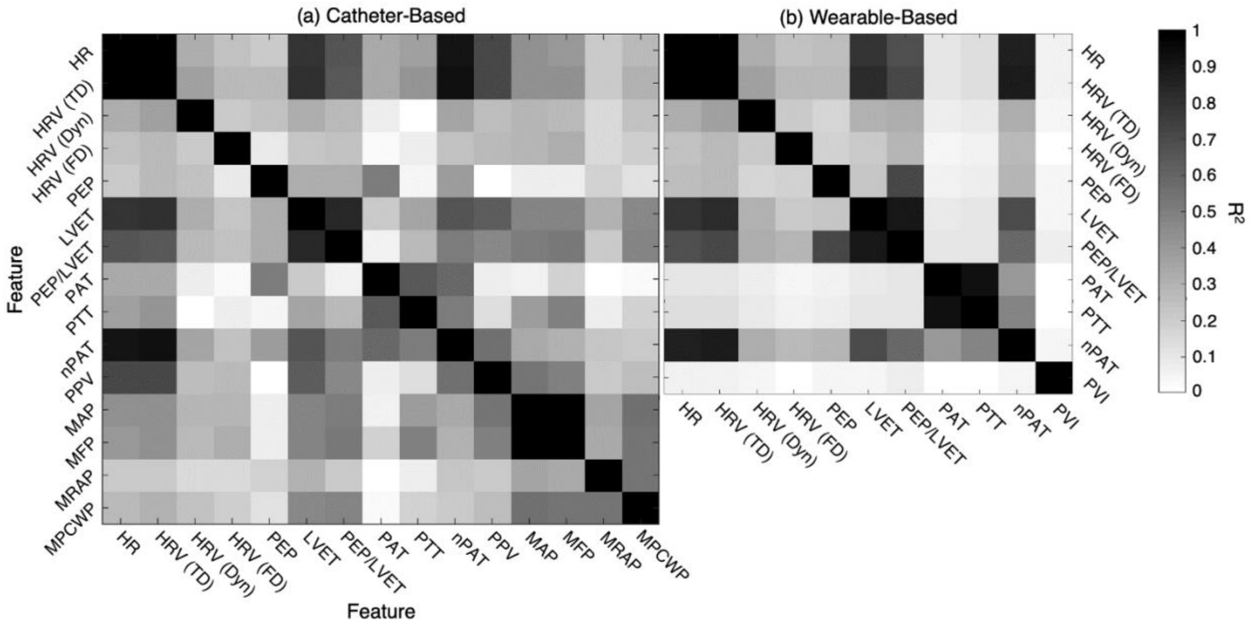

Supp. Fig. S4. Correlation of Physiological Features. Coefficient of determination (R2) computed for each pair of features from all data collected from all animal subjects during the protocol. The analysis was performed separately for both the (**a**) catheter-based and (**b**) wearable system.

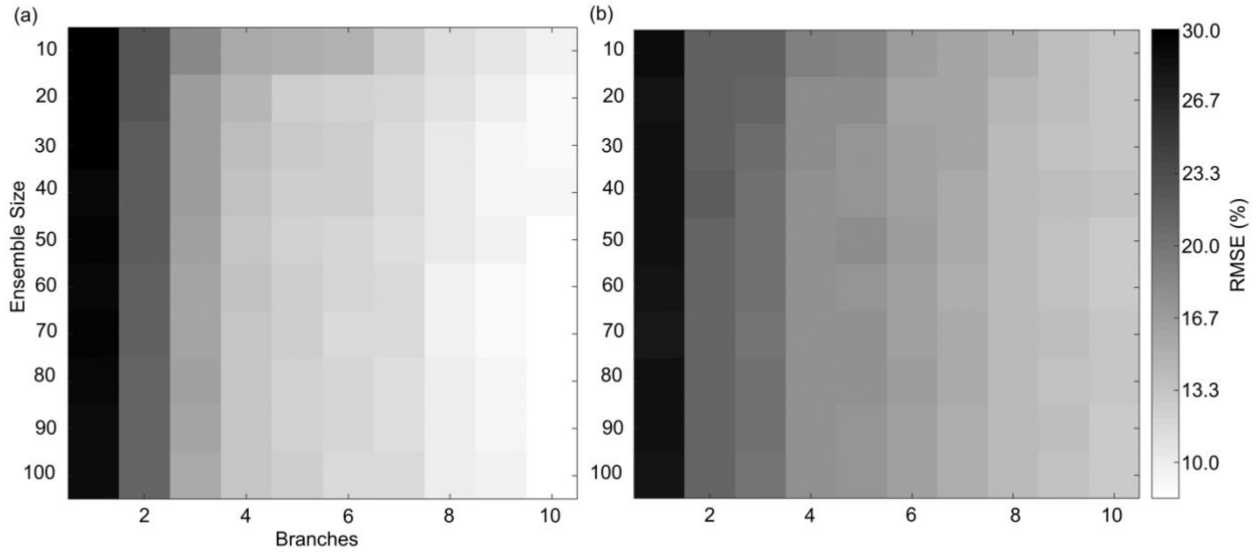

Supp. Fig. S5. Hyperparameter Effect on BVS Estimation. RMSE between ground-truth and estimated blood volume status for models trained using (**a**) catheter-based and (**b**) wearable systems averaged across animal subjects. The RMSE is plotted against the size of the random forest ensemble and maximum depth of each regression tree.

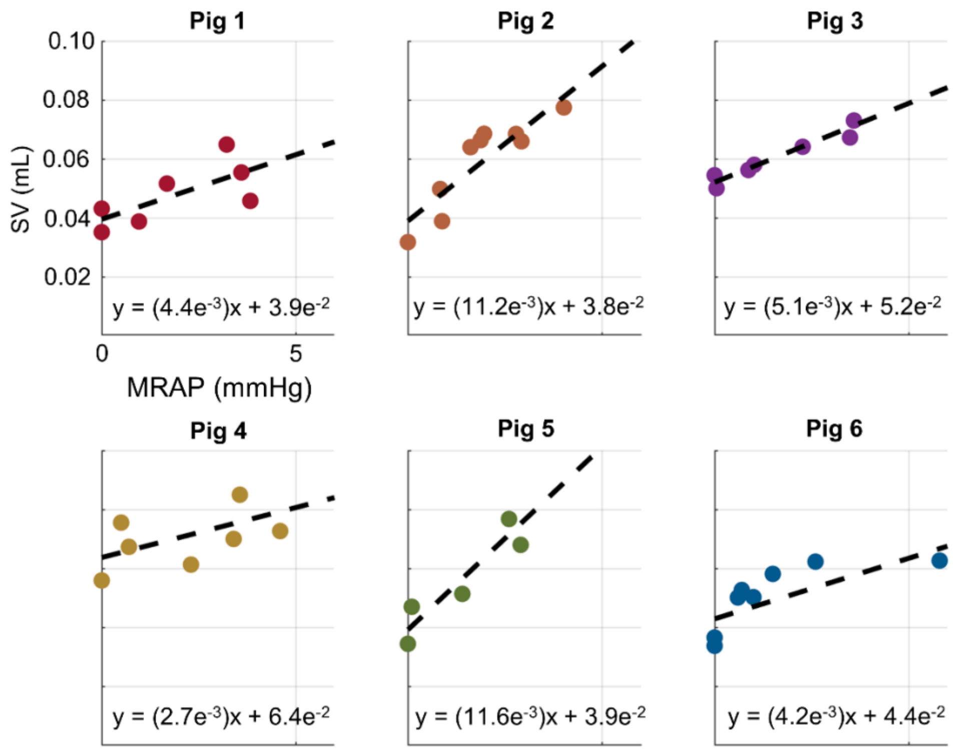

Supp. Fig. S6. SV Response to Preload Changes. Scatterplots of SV and preload (mean right atrial pressure) recorded during the exsanguination protocol for each animal subject. Best-fit lines (black, dashed) and their corresponding equations are overlaid.

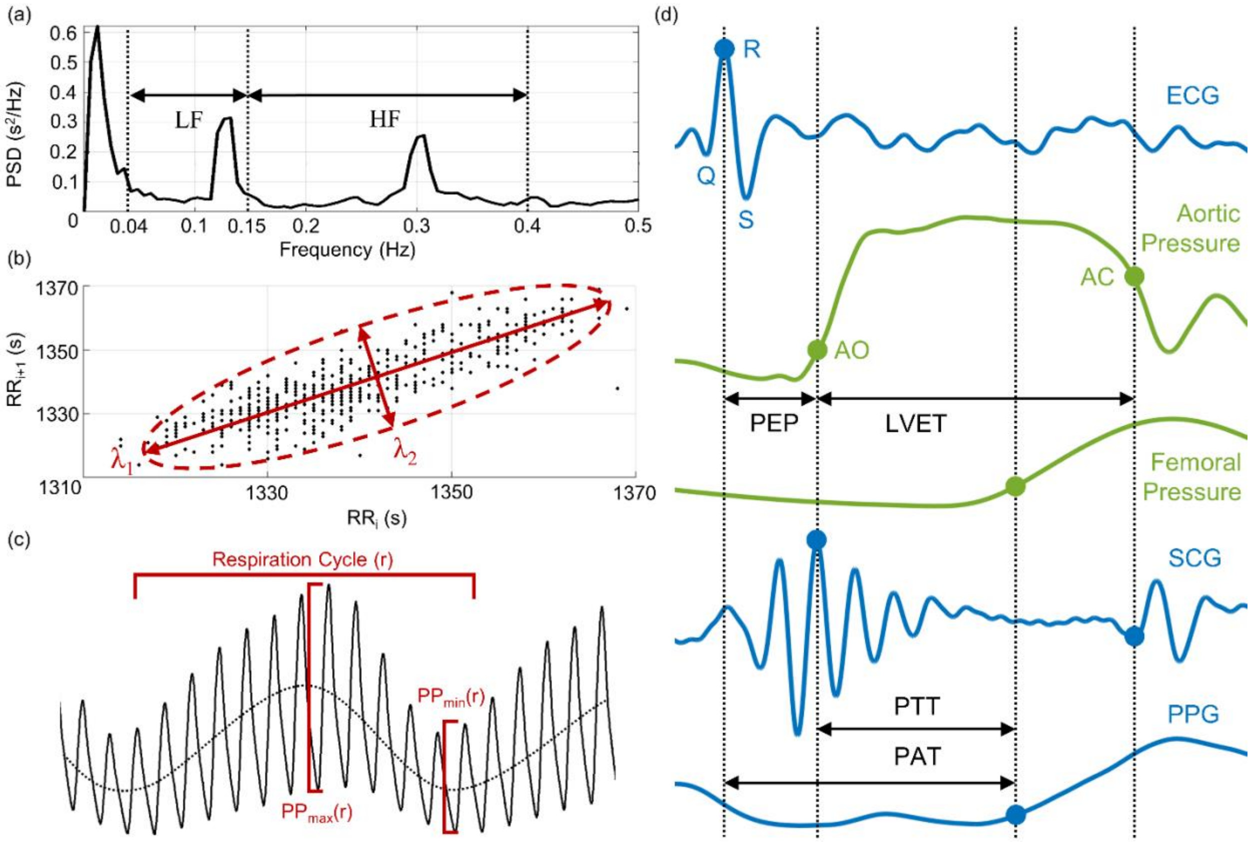

Supp. Fig. S7. Derivation of Physiological Features. (**a**) Sample frequency spectrum for a 500-heartbeat interval of RRIs at rest. LF: low-frequency, HF: high-frequency. (**b**) Sample Poincaré plot for 500-hearbeat interval from (**a**). λ_1 and λ_2 represent the first and second eigenvalues of the covariance matrix respectively. (**c**) Extraction of PP_{min} and PP_{max} for a sample femoral artery pressure waveform (black, solid) over a single respiration cycle. Mean femoral artery pressure is overlaid (black, dotted). (**d**) Cardiac timing interval extraction from wearable- (blue) and catheter-based (green) signals for a sample heartbeat. PSD: power spectral density.

REFERENCES AND NOTES

1. B. J. Eastridge, M. Hardin, J. Cantrell, L. Oetjen-Gerdes, T. Zubko, C. Mallak, C. E. Wade, J. Simmons, J. Mace, R. Mabry, R. Bolenbaucher, L. H. Blackbourne, Died of wounds on the battlefield: Causation and implications for improving combat casualty care. *J. Trauma* **71**, S4–S8 (2011).

2. H. R. Champion, R. F. Bellamy, C. P. Roberts, A. Leppaniemi, A profile of combat injury. *J. Trauma* **54**, S13–S19 (2003).

3. V. A. Convertino, G. Grudic, J. Mulligan, S. Moulton, Estimation of individual-specific progression to impending cardiovascular instability using arterial waveforms. *J. Appl. Physiol.* **115**, 1196–1202 (2013).

4. T. E. Schlotman, K. R. Lehnhardt, A. F. Abercromby, B. D. Easter, M. E. Downs, L. T. C. K. S. Akers, V. A. Convertino, Bridging the gap between military prolonged field care monitoring and exploration spaceflight: The compensatory reserve. *NPJ Microgravity* **5**, 29 (2019).

5. U. R. Acharya, K. P. Joseph, N. Kannathal, C. M. Lim, J. S. Suri, Heart rate variability: A review. *Med. Biol. Eng. Comput.* **44**, 1031–1051 (2006).

6. K. Lee, X. Ni, J. Y. Lee, H. Arafa, D. J. Pe, S. Xu, R. Avila, M. Irie, J. H. Lee, R. L. Easterlin, D. H. Kim, H. U. Chung, O. O. Olabisi, S. Getaneh, E. Chung, M. Hill, J. Bell, H. Jang, C. Liu, J. B. Park, J. Kim, S. B. Kim, S. Mehta, M. Pharr, A. Tzavelis, J. T. Reeder, I. Huang, Y. Deng, Z. Xie, C. R. Davies, Y. Huang, J. A. Rogers, Mechano-acoustic sensing of physiological processes and body motions via a soft wireless device placed at the suprasternal notch. *Nat. Biomed. Eng.* **4**, 148–158 (2019).

7. M. Etemadi, O. T. Inan, Wearable ballistocardiogram and seismocardiogram systems for health and performance. *J. Appl. Physiol.* **124**, 452–461 (2017).

8. R. S. Crow, P. Hannan, D. Jacobs, L. Hedquist, D. M. Salerno, Relationship between seismocardiogram and echocardiogram for events in the cardiac cycle. *Am. J. Noninvas. Card.* **8**, 39–46 (1994).

9. J. Allen, Photoplethysmography and its application in clinical physiological measurement. *Physiol. Meas.* **28**, R1–R39 (2007).

10. R. Mukkamala, J.-O. Hahn, O. T. Inan, L. K. Mestha, C.-S. Kim, H. Töreyin, S. Kyal, Toward ubiquitous blood pressure monitoring via pulse transit time: Theory and practice. *IEEE Trans. Biomed. Eng.* **62**, 1879–1901 (2015).

11. G. Natalini, A. Rosano, M. E. Franceschetti, P. Facchetti, A. Bernardini, Variations in arterial blood pressure and photoplethysmography during mechanical ventilation. *Anesth. Analg.* **103**, 1182–1188 (2006).

12. K. H. Shelley, Photoplethysmography: Beyond the calculation of arterial oxygen saturation and heart rate. *Anesth. Analg.* **105**, S31–S36 (2007).

13. O. Gödje, M. Peyerl, T. Seebauer, P. Lamm, H. Mair, B. Reichart, Central venous pressure, pulmonary capillary wedge pressure and intrathoracic blood volumes as preload indicators in cardiac surgery patients. *Eur. J. Cardiothorac. Surg.* **13**, 533–539 (1998).

14. M. Lichtwarck-Aschoff, R. Beale, U. J. Pfeiffer, Central venous pressure, pulmonary artery occlusion pressure, intrathoracic blood volume, and right ventricular end-diastolic volume as indicators of cardiac preload. *J. Crit. Care* **11**, 180–188 (1996).

15. K. Sørensen, S. E. Schmidt, A. S. Jensen, P. Søgaard, J. J. Struijk, Definition of fiducial points in the normal seismocardiogram. *Sci. Rep.* **8**, 15455 (2018).

16. S. Loukogeorgakis, R. Dawson, N. Phillips, C. N. Martyn, S. E. Greenwald, Validation of a device to measure arterial pulse wave velocity by a photoplethysmographic method. *Physiol. Meas.* **23**, 581–596 (2002).

17. J. T. Howard, J. C. Janak, C. Hinojosa-Laborde, V. A. Convertino, Specificity of compensatory reserve and tissue oxygenation as early predictors of tolerance to progressive reductions in central blood volume. *Shock* **46**, 68–73 (2016).

18. L. Breiman, Random forests, *Mach. Learn.* **45**, 5–32 (2001).

19. U. Grömping, Variable importance assessment in regression: Linear regression versus random forest. *Am. Stat.* **63**, 308–319 (2009).

20. S. S. Ahmed, G. E. Levinson, C. J. Schwartz, P. O. Ettinger, Systolic time intervals as measures of the contractile state of the left ventricular myocardium in man. *Circulation* **46**, 559–571 (1972).

21. C. L. Garrard Jr., A. M. Weissler, H. T. Dodge, The relationship of alterations in systolic time intervals to ejection fraction in patients with cardiac disease. *Circulation* **42**, 455–462 (1970).

22. C. Hinojosa-Laborde, J. T. Howard, J. Mulligan, G. Z. Grudic, V. A. Convertino, Comparison of compensatory reserve during lower-body negative pressure and hemorrhage in nonhuman primates. *Am. J. Physiol. Regul. Integr. Comp. Physiol.* **310**, R1154–R1159 (2016).

23. M. R. Pinsky, Cardiopulmonary interactions: Physiologic basis and clinical applications, *Ann. Am. Thorac. Soc.* **15**, S45–S48 (2018).

24. J. Kranjec, S. Beguš, G. Geršak, J. Drnovšek, Non-contact heart rate and heart rate variability measurements: A review. *Biomed. Signal Process. Control* **13**, 102–112 (2014).

25. A. Taebi, B. E. Solar, A. J. Bomar, R. H. Sandler, H. A. Mansy, Recent advances in seismocardiography. *Vibration* **2**, 64–86 (2019).

26. D. Shao, Y. Yang, C. Liu, F. Tsow, H. Yu, N. Tao, Noncontact monitoring breathing pattern, exhalation flow rate and pulse transit time. *IEEE Trans. Biomed. Eng.* **61**, 2760–2767 (2014).

27. H. Schou, V. P. de Sá, M. Sigurdardottir, R. Roscher, C. Jonmarker, O. Werner, Circulatory effects of hypoxia, acute normovolemic hemodilution, and their combination in anesthetized pigs. *Anesthesiology* **84**, 1443–1454 (1996).

28. J. A. Lieberman, R. B. Weiskopf, S. D. Kelley, J. Feiner, M. Noorani, J. Leung, P. Toy, M. Viele, Critical oxygen delivery in conscious humans is less than 7.3 ml O^2 kg^{-1} min^{-1}. *Anesthesiology* **92**, 407–407 (2000).

29. A. C. Ertl, A. Diedrich, S. R. Raj, Techniques used for the determination of blood volume. *Am. J. Med. Sci.* **334**, 32–36 (2007).

30. C. Hinojosa-Laborde, R. E. Shade, G. W. Muniz, C. Bauer, K. A. Goei, H. F. Pidcoke, K. K. Chung, A. P. Cap, V. A. Convertino, Validation of lower body negative pressure as an experimental model of hemorrhage. *J. Appl. Physiol.* **116**, 406–415 (2013).

31. W. Ganz, R. Donoso, H. S. Marcus, J. S. Forrester, H. J. Swan, A new technique for measurement of cardiac output by thermodilution in man. *Am. J. Cardiol.* **27**, 392–396 (1971).

32. M. M. H. Shandhi, B. Semiz, S. Hersek, N. Goller, F. Ayazi, O. T. Inan, Performance analysis of gyroscope and accelerometer sensors for seismocardiography-based wearable pre-ejection period estimation. *IEEE J. Biomed. Health Inform.* **23**, 2365–2374 (2019).

33. L. Breiman, Bagging predictors. *Mach. Learn.* **24**, 123–140 (1996).

34. P. Probst, M. N. Wright, A.-L. Boulesteix, Hyperparameters and tuning strategies for random forest. *Wiley Interdisc. Rev. Data Mining Knowl. Discov.* **9**, e1301 (2019).

35. C. M. Bishop, *Pattern Recognition and Machine Learning* (Springer, 2006).

36. L. S. Lilly, *Pathophysiology of Heart Disease: A Collaborative Project of Medical Students and Faculty* (Lippincott Williams & Wilkins, 2012).

37. D. D. Glower, J. A. Spratt, N. D. Snow, J. S. Kabas, J. W. Davis, C. O. Olsen, G. S. Tyson, D. C. Sabiston Jr., J. S. Rankin, Linearity of the Frank-Starling relationship in the intact heart: The concept of preload recruitable stroke work. *Circulation* **71**, 994–1009 (1985).

38. P. Castiglioni, A. Faini, G. Parati, M. Di Rienzo, Wearable seismocardiography, in *2007 29th Annual International Conference of the IEEE Engineering in Medicine and Biology Society* (2007).

39. A. A. Alian, K. H. Shelley, Photoplethysmography. *Best Pract. Res. Clin. Anaesthesiol.* **28**, 395–406 (2014).

40. W. H. Cooke, V. A. Convertino, Heart rate variability and spontaneous baroreflex sequences: Implications for autonomic monitoring during hemorrhage. *J. Trauma* **58**, 798–805 (2005).

41. E. Salomão Jr., D. A. Otsuki, A. L. Correa, D. T. Fantoni, F. dos Santos, M. C. Irigoyen, J. O. C. Auler Jr., Heart rate variability analysis in an experimental model of hemorrhagic shock and resuscitation in pigs. *PLOS ONE* **10**, e0134387 (2015).

42. P. W. Kamen, H. Krum, A. M. Tonkin, Poincaré plot of heart rate variability allows quantitative display of parasympathetic nervous activity in humans. *Clin. Sci.* **91**, 201–208 (1996).

43. K. C. Bilchick, R. D. Berger, Heart rate variability. *J. Cardiovasc. Electrophysiol.* **17**, 691–694 (2006).

44. P. E. Marik, X. Monnet, J.-L. Teboul, Hemodynamic parameters to guide fluid therapy. *Ann. Intensive Care* **1**, 1 (2011).

45. F. Michard, J.-L. Teboul, Predicting fluid responsiveness in ICU patients: A critical analysis of the evidence. *Chest* **121**, 2000–2008 (2002).

46. M. Cannesson, O. Desebbe, P. Rosamel, B. Delannoy, J. Robin, O. Bastien, J.-J. Lehot, Pleth variability index to monitor the respiratory variations in the pulse oximeter plethysmographic waveform amplitude and predict fluid responsiveness in the operating theatre. *Br. J. Anaesth.* **101**, 200–206 (2008).

47. M. Zimmermann, T. Feibicke, C. Keyl, C. Prasser, S. Moritz, B. M. Graf, C. Wiesenack, Accuracy of stroke volume variation compared with pleth variability index to predict fluid responsiveness in mechanically ventilated patients undergoing major surgery. *Eur. J. Anaesthesiol.* **27**, 555–561 (2010).

48. D. B. Newlin, R. W. Levenson, Pre-ejection period: Measuring beta-adrenergic influences upon the heart. *Psychophysiology* **16**, 546–552 (1979).

49. G. S. H. Chan, P. M. Middleton, B. G. Celler, L. Wang, N. H. Lovell, Change in pulse transit time and pre-ejection period during head-up tilt-induced progressive central hypovolaemia. *J. Clin. Monit. Comput.* **21**, 283–293 (2007).

50. J. Zia, J. Kimball, M. H. Shandhi, O. T. Inan, Automated Identification of Persistent Time-Domain Features in Seismocardiogram Signals, in *2019 IEEE EMBS International Conference on Biomedical & Health Informatics (BHI)* (2019).

51. T. B. Graboys, F. J. Forlini Jr., E. D. Michaelson, Systolic time intervals during lower body negative pressure. *J. Appl. Physiol.* **37**, 329–332 (1974).

52. K. Tavakolian, G. A. Dumont, G. Houlton, A. P. Blaber, Precordial vibrations provide noninvasive detection of early-stage hemorrhage. *Shock* **41**, 91–96 (2014).

53. M. Vettorello, S. Sher, S. Santambrogio, A. Calini, F. Tardini, M. Lippi, R. Fumagalli, Validation of a novel index of hemorrhage using a lower body negative pressure shock model. *Minerva Anestesiol.* **82**, 839–849 (2016).

54. U. J. Pfeiffer, M. Perker, J. Zeravik, G. Zimmermann, Sensitivity of central venous pressure, pulmonary capillary wedge pressure, and intrathoracic blood volume as indicators for acute and chronic hypovolemia, in *Practical Applications of Fiberoptics in Critical Care Monitoring* (Springer, 1990), pp. 25–31.

55. G. Gutierrez, H. D. Reines, M. E. Wulf-Gutierrez, Clinical review: Hemorrhagic shock. *Crit. Care* **8**, 373–381 (2004).
